# DNA Methylation in Thyroid Tumorigenesis

**DOI:** 10.3390/cancers3021732

**Published:** 2011-03-29

**Authors:** Josena K. Stephen, Dhananjay Chitale, Vinod Narra, Kang Mei Chen, Raja Sawhney, Maria J. Worsham

**Affiliations:** 1. Department of Otolaryngology/Head and Neck Surgery, Henry Ford Hospital, Detroit, MI 48202, USA; E-Mails: kchen1@hfhs.org (K.M.C.); sawhneyraja@gmail.com (R.S.); mworsha1@hfhs.org (M.J.W.); 2. Department of Pathology, Henry Ford Hospital, Detroit, MI 48202, USA; E-Mail: DChital1@hfhs.org; 3. Essex Surgical Associates, PC, Beverly, MA 01915, USA; E-Mail: vnarra@partners.org

**Keywords:** papillary thyroid cancer, follicular thyroid cancer, hypermethylation, *NIS*, *CASP8*, *RASSF1*

## Abstract

Thyroid cancer is the most common endocrine cancer with 1,690 deaths each year. There are four main types of which the papillary and follicular types together account for >90% followed by medullary cancers with 3% to 5% and anaplastic carcinomas making up <3%. Epigenetic events of DNA hypermethylation are emerging as promising molecular targets for cancer detection. Our immediate and long term goal is to identify DNA methylation markers for early detection of thyroid cancer. This pilot study comprised of 21 patients to include 11 papillary thyroid cancers (PTC), 2 follicular thyroid cancers (FTC), 5 normal thyroid cases, and 3 hyperthyroid cases. Aberrant promoter methylation was examined in 24 tumor suppressor genes using the methylation specific multiplex ligation-dependent probe amplification (MS-MLPA) assay and in the *NIS* gene using methylation-specific PCR (MSP). The frequently methylated genes were *CASP8* (17/21), *RASSF1* (16/21) and *NIS* (9/21). In the normal samples, *CASP8, RASSF1* and *NIS* were methylated in 5/5, 4/5 and 1/5 respectively. In the hyperthyroid samples, *CASP8, RASSF1* and *NIS* were methylated in 3/3, 2/3 and 1/3 respectively. In the thyroid cancers, *CASP8*, *RASSF1*, and *NIS* were methylated in 9/13, 10/13, and 7/13 respectively. *CASP8, RASSF1* and *NIS* were also methylated in concurrently present normal thyroid tissue in 3/11, 4/11 and 3/11 matched thyroid cancer cases (matched for presence of both normal thyroid tissue and thyroid cancer), respectively. Our data suggests that aberrant methylation of *CASP8*, *RASSF1*, and *NIS* maybe an early change in thyroid tumorigenesis regardless of cell type.

## 1. Introduction

The National Cancer Institute [1] estimated that in 2010 there would be 44,670 new cases of thyroid cancer (TC) with 1,690 deaths. Compared to other adult cancers, TC tends to occur in younger people between the ages of 20 and 60 years [2]. It is three times more common in women than men [2], has the fastest rising incidence rates in women, and the second fastest in men with an annual percentage change of approximately 5%, making TC the sixth most common cancer in women [3]. Reasons for this trend have been attributed to improvement in imaging (ultrasound technology) that is allowing the identification of ever smaller thyroid nodules. However, with this gain in detection, determining which benign nodules (adenomas) will progress to cancer cannot be determined on the basis of histology alone, underscoring the need for genetic markers of early detection for TC.

There are four main types of thyroid cancers: papillary, follicular, medullary and anaplastic. The papillary and follicular types, also known as differentiated thyroid cancers, account for >90% of thyroid carcinomas, followed by medullary cancers with 3–5% and anaplastic carcinomas with <3% [4]. Papillary thyroid cancers (PTC) are the most common and have the best prognosis. Follicular thyroid carcinomas (FTC) are the next most common type of thyroid cancer and have a slightly worse prognosis than PTC. The cells in follicular carcinomas can accumulate radioactive iodine which can be used in its treatment. Hürthle cell carcinoma, also known as oxyphil cell carcinoma, is a variant of follicular carcinoma but has a worse outcome. It is less likely to concentrate radioactive iodine [4] making it harder to detect and treat. Besides age, other risk factors for developing thyroid cancer include diets low in iodine, radiation exposure and certain hereditary conditions like familial medullary thyroid carcinoma, multiple endocrine neoplasia type 2A (MEN-2A) and multiple endocrine neoplasia type 2B (MEN-2B).

Until recently, thyroid cancer has been largely ignored in the US because the majority of thyroid cancer cases have very good prognosis, with five-year survival rates of approximately 96% when submitted to timely and appropriate treatment [3,5]. However, those diagnosed at late stages and presenting with advanced diseases have a devastating five-year survival rate of under 60% [6]. The recurrence rate of thyroid cancer is high (30%) [7], and only one third of patients with distant metastases respond to radioactive iodine (^131^I) therapy with complete remission [8]. Thus, despite advances on scientific and clinical fronts, advanced thyroid cancers remain incurable. The present challenge is to obtain a highly accurate diagnostic test for early stage thyroid cancers and effective molecular-directed therapies for advanced thyroid cancer.

Early detection of cancers before metastasis is important for patients and clinicians as it improves prognosis, patient quality of life and provides additional treatment options. One of the best ways for early detection of cancer is through the use biomarkers. These can be DNA, mRNA, proteins, metabolites, or processes such as apoptosis, angiogenesis or proliferation [9]. These markers of early tumor development can be produced by either the tumor or by other tissues, so they are present in all types of body fluids, tissues or cell lines. Tumor markers include hormones, functional subgroups of proteins (like enzymes, glycoproteins, and receptors), molecular markers (such as genetic mutations, amplifications or translocations) [10] and epigenetic markers (like tumor suppressor gene hypermethylation) [11]. Beyond their screening and diagnostic value, biomarkers can be used to estimate tumor volume, evaluate response to treatment, assess disease recurrence, or as prognostic indicators of disease progression [10].

Over the past 15 years, the application of molecular technologies has focused on genetic events. New approaches are necessary to identify potential novel diagnostic and prognostic markers, which would allow accurate early diagnosis, with personalized clinical management and surveillance. Epigenetic silencing through aberrant DNA methylation of tumor suppressor genes has been reported in TC [11]. Methylation of *TSHR, ECAD, NIS-L, ATM* and *DAPK* are frequent in PTC [12]. Increased methylation of several tumor suppressor genes (*TIMP3, SLC5A8, DAPK*, and *RARB2*) is associated with features of PTC aggressiveness [11,13]. Hypermethylation of *RASSF1A*, a known tumor suppressor gene, has been described in 75% (9 of 12) of follicular thyroid cancers as well as in a smaller percentage of benign adenomas (44%), and papillary thyroid cancers (20%) [14] indicating that this may be an early step in follicular cell derived thyroid tumorigenesis.

Our immediate and long-term goal is to identify DNA methylation markers for early detection of thyroid cancer. In this exploratory study, we examined promoter hypermethylation in 24 tumor suppressor genes using the methylation-specific multiplex ligation-dependent probe amplification (MS-MLPA) assay and in the *NIS* gene using methylation-specific PCR (MSP).

## 2. Results and Discussion

In this study, *CASP8 and RASSF1* were the only two genes found to be aberrantly methylated from the MS-MLPA panel of 24 tumor suppressor genes. Additionally, the *NIS* gene was selected to be examined by MSP as it is not present in the MS-MLPA gene panel, is known to be methylated in PTC [12], and is a thyroid-specific gene that plays a role in iodine uptake and normal thyroid cell function [15-17]. Aberrant methylation of *CASP8* was most frequent occurring in 17/21 samples, followed by *RASSF1* in 16/21 and *NIS* in 9/21. Methylation of *CASP8* was observed in all 5 normal samples, all 3 hyperthyroid samples and in 3/11 concurrent thyroid cancer with normal thyroid lesions. This was followed by *RASSF1* in 4/5 normal samples, 2/3 hyperthyroid samples and in 4/11 concurrent thyroid cancer with normal thyroid lesions. *NIS* methylation occurred in 1 normal thyroid sample, 1 hyperthyroid sample and in 3/11 concurrent thyroid cancer with normal thyroid lesions.

Proper management of TC depends on accurate pathological diagnosis and although most thyroid nodules are benign, distinguishing thyroid cancer from benign lesions is crucial for appropriate treatment and follow-up. Fine-needle aspiration (FNA) biopsy is the standard diagnostic tool for preoperative diagnosis [18]. The FNA biopsy allows diagnosis of the nature of thyroid nodules in a majority of cases, but it has some limitations, especially in the presence of follicular lesions where it can be difficult to distinguish between a follicular adenoma (FA) and FTC because they are cytologically indistinguishable [19]. About 20% of FNA biopsies performed for nodular thyroid lesions in USA are reported as inconclusive or suspicious for malignancy [19]. A majority of these inconclusive cases (70%) [20] are benign. A single false-negative FNA can delay surgical treatment by 28 months even with clinical evidence suggesting malignancy, leading to higher rates of vascular and capsular invasion and persistent disease at follow up which is in the advanced stage making it incurable [21]. Even amongst PTC, where diagnosis is straightforward on FNA, the tall cell variant (TCV), which has a worse prognosis, can be misdiagnosed [22,23]. FNA has been found to be less sensitive in detecting FTC and Hürthle cell carcinomas when compared to PTC [21]. The diagnosis of PTC, MTC and anaplastic thyroid cancer by FNA biopsy is straightforward in that it is based on nuclear changes that are specific to each of these types of cancers, whereas the diagnosis of FA and FTC is based on the presence of capsular and vascular invasion which cannot be seen on FNA [24]. Only on surgical removal can FA be differentiated from FTC. Hence, there is a need for reliable, novel preoperative markers to help differentiate benign and malignant thyroid nodules.

In this exploratory study methylation of *CASP8* is the most frequent (17/21 cases) and appears to be an early change since it was present in all 5 normal samples, all 3 hyperthyroid samples and in 3/11 concurrent thyroid cancer with normal thyroid lesions.

Methylation of *CASP8* in thyroid cancer is relatively new. It has been reported in childhood medulloblastoma and neuroblastoma [25,26], neuroendocrine lung tumors [27] and breast cancer [28]. The gene is located on chromosome 2q33, encodes a member of the caspase family, Caspase-8, which is an apical caspase acting in the death receptor–ligand interaction–induced apoptotic process [29]. The absence or downregulation of CASP8 may be attributed to epigenetic changes, such as hypermethylation, or mutations [30,31] The promoter region has binding sites for p53, nuclear factor-κB (NF-κB), AP-1, SP-1, IRF-1, and Ets-like transcription factors [32]. CASP8 functions both as a key molecule for apoptosis as well as a selective signal transducer [33]. As an important initiator of apoptosis [34], loss or reduced expression supports its role in tumorigenesis. The cytotoxic drugs 5-fluorouracil (5-Fu) and methotrexate, used to treat some cancers, have been shown to upregulate *CASP8* and induce cell apoptosis in multiple cancers including breast cancer [35,36]. A study by Wu *et al.* [28] found that breast cancer cells treated with 5-Fu induced cell apoptosis by demethylation of the *CASP8* promoter region resulting in a significant increase in its mRNA expression.

*RASSF1* was the next most frequently methylated gene, detected in 16 of the 21 cases. Among the 16 cases, 10 cases demonstrated *RASSF1* methylation as an early change. It was methylated in 4/5 normal samples, 2/3 hyperthyroid samples and in 4/11 concurrent thyroid cancer with normal thyroid lesions. *RASSF1* encodes a signaling protein that functions through a pathway involving Ras which is a component of the phosphatidylinositol 3-kinase (PI3K)/Akt pathway [11]. Mutational inactivation of this gene is very rare (<2%), and the main mechanism of its inactivation is through promoter methylation and LOH [37]. *RASSF1* has been found to be methylated in benign thyroid lesions and thyroid cancers, including PTC, FTC, and ATC [14,37].

Methylation of thyroid-specific genes, such as those for sodium/iodide symporter *(NIS)* and thyroid-stimulating hormone receptor, is a cause for the failure of clinical radioiodine treatment of thyroid cancer [38,39]. Methylation of *NIS* has severe treatment implications in TC. The function of the thyroid is to produce thyroid hormones, through the uptake and concentration of iodine, to meet the body's normal metabolic needs. Thyroid specific genes, thyroid peroxidase (*TPO*), thyroglobulin [40], TSH receptor (*TSHR*) and *NIS*, all play a role in iodine uptake and normal thyroid cell function [15-17]. The cornerstone of follow-up and treatment of patients with thyroid cancer is by the measurement of iodide uptake by thyroid cancer cells. However, radioiodide uptake is found only in about 67% of patients with persistent or recurrent disease. Several studies have demonstrated a decrease in or a loss of NIS expression in primary human thyroid carcinomas [41,42]. Immunohistochemical studies have confirmed this considerably decreased expression of the NIS protein in thyroid cancer tissues, suggesting that low expression of NIS may represent an early abnormality in the pathway of thyroid cell transformation, rather than being a consequence of cancer progression [43]. *NIS* methylation has been reported in advanced stage PTC but was not detected in adjacent benign tissue [12]. In this study, *NIS* methylation occurred in 1 normal thyroid sample, 1 hyperthyroid sample and in 3/11 concurrent thyroid cancer with normal thyroid lesions.

A distinct advantage of MS-MLPA is the ability to examine aberrant promoter methylation in multiple cancer genes in a single assay using formalin-fixed paraffin embedded tissue. This is because probes added to the samples are amplified and quantified instead of target nucleic acids. The probe target sequences are small (50-70 nucleotides), making it ideal for fragmented formalin-fixed paraffin embedded tissue DNA. When compared to MSP, the MS-MLPA assay requires minimal sample preparation and very small quantities of DNA (20 ng). It is extremely easy to perform and is an inexpensive one-tube assay compared to array based technologies. MS-MLPA has been validated in other studies by our group [44,45] with a high degree of sensitivity and specificity as well as by MRC Holland [46], the makers of MS-MLPA. MRC Holland demonstrated the sensitivity of MS-MLPA by analyzing human sample DNA methylated in vitro which resulted in amplification products for all probes. Specificity was demonstrated by noting that all MS-MLPA probes that recognized a *Hha*I site within a CpG island did not produce amplification products after *Hha*I digestion. MS-MLPA profiling of multiple genes for aberrantly methylated promoter regions is a valuable screening tool to determine frequency and pattern of promoter methylation in neoplasia. These epigenetic signatures, upon subsequent validation as diagnostic or prognostic biomarkers, can become reduced to a more definitive candidate gene panel of only a few key genes.

*CASP8*, *RASSF1*and *NIS*, were found to be consistently methylated in normal thyroid, hyperthyroid lesions, as well as in those thyroid cancer cases with matched normal and tumor blocks from the same biopsy specimen and likely represent early changes in thyroid tumorigenesis.

## 3. Experimental Section

### 3.1. Cohort

The cohort of 21 patients consisted of 11 papillary thyroid cancers (PTC), 2 follicular thyroid cancers (FTC), 5 normal thyroid cases and 3 hyperthyroid cases. Of the 2 FTC, one was a poorly differentiated follicular (insular) carcinoma and the other was a Hürthle cell carcinoma. Thyroid cancer specimens were matched for normal and tumor lesions in 11 of the 13 cases where normal and tumor blocks were taken from the same biopsy specimen. The five histologically normal thyroid tissues were from patients who had their thyroid removed at the time of surgery for laryngeal cancers. The 3 cases of hyperthyroidism were due to Graves' disease.

### 3.2. DNA Extraction

Whole 5 micron tissue sections or microdissected thyroid lesions and adjacent normal when present were processed for DNA extraction as previously described [47].

### 3.3. Methylation-Specific Multiplex Ligation-dependent Probe Amplification (MS-MLPA) Assay

MS-MLPA, a modification of the conventional MLPA assay, allows for the simultaneous detection of changes in methylation status as well as copy number changes of approximately 41 different DNA sequences in a single reaction requiring only 20 ng of human DNA [46,48,49].

**Figure 1.**
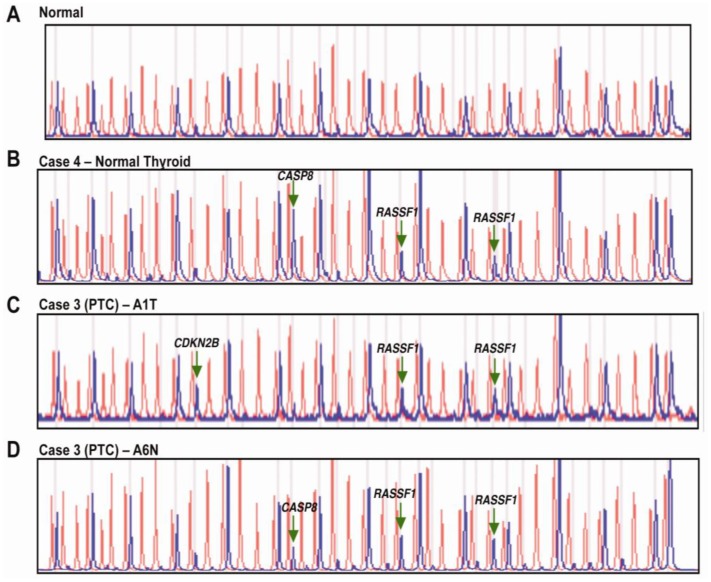
(**A**) Normal control DNA sample with 41 individual peaks (red) in the absence of *Hha*I and 15 separate peaks (blue) in the presence of *Hha*I. (**B**) Normal thyroid sample with methylation of *CASP8* and *RASSF1*. (**C**) Papillary thyroid cancer (Case 3 - tumor block) with methylation of *CDKN2B* and *RASSF1*. (**D**) Papillary thyroid cancer (Case 3 -normal block) with methylation of *CASP8* and *RASSF1*. (PTC – papillary thyroid cancer, A1T – block A1 tumor, A6N – block A6 normal).

**Figure 2.**
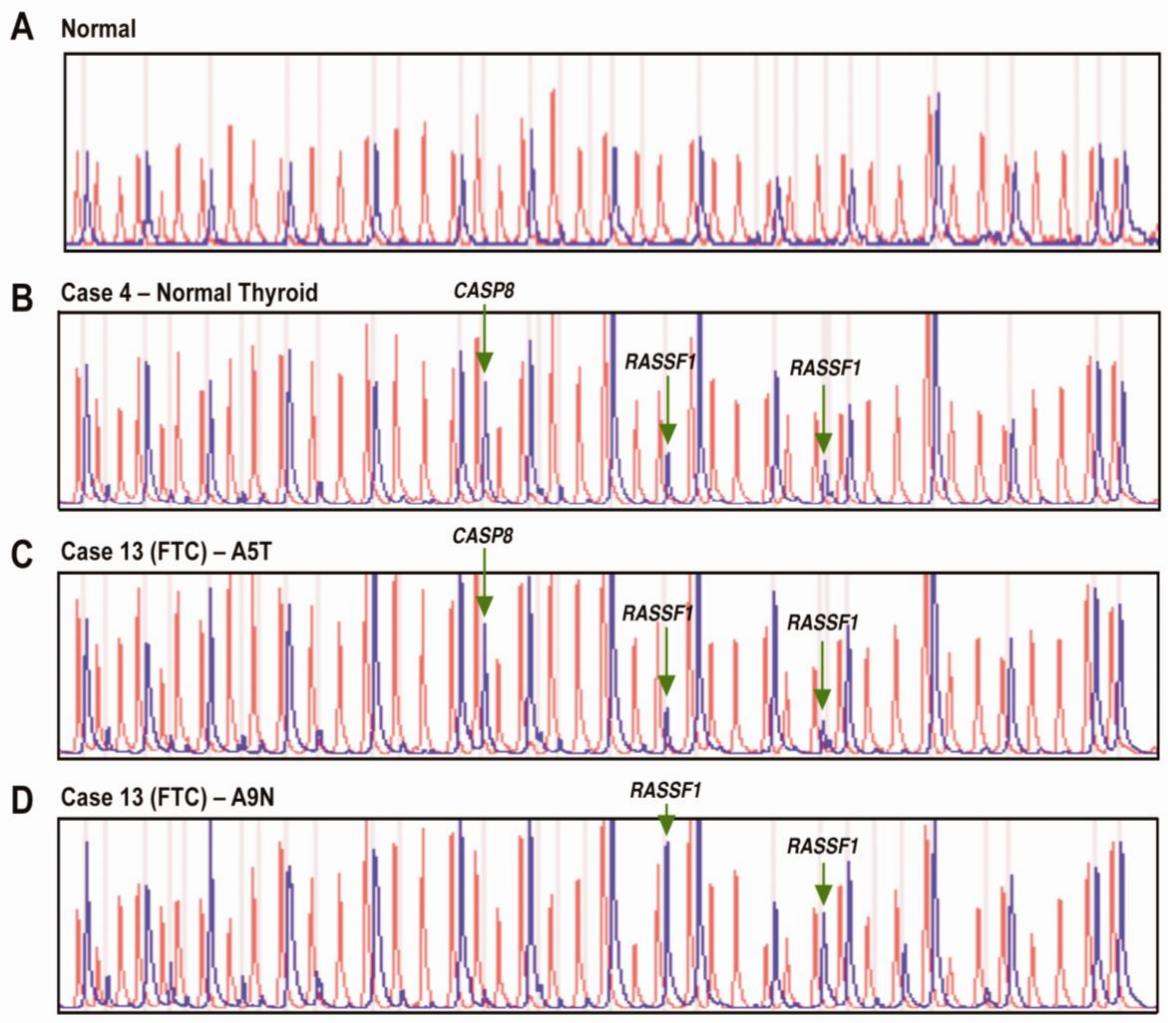
(**A**) Normal control DNA sample with 41 individual peaks (red) in the absence of *Hha*I and 15 separate peaks (blue) in the presence of *Hha*I. (**B**) Normal thyroid sample with methylation of *CASP8* and *RASSF1*. (**C**) Follicular thyroid cancer (Case 13 - tumor block) with methylation of *CASP8* and *RASSF1*. (**D**) Follicular thyroid cancer (Case 13 -normal block) with methylation of *RASSF1*. (FTC – follicular thyroid cancer, A5T – block A5 tumor, A9N – block A9 normal).

Briefly, the MS-MLPA panel in the presence of *Hha*I detects aberrant promoter hypermethylation by taking advantage of a *Hha*I site in the gene probes of interest. The control gene probes, without a *Hha*I site, serve as undigested controls. A normal control DNA sample will generate 41 individual peaks for all probes in the absence of *Hha*I and 15 separate peaks in the presence of *Hha*I (Figure 1A and Figure 2A). Normal controls for methylation assays are run using DNA from paraffin-embdedded squamous epithelium from individuals with no evidence of cancer.

### 3.4. Gene Probe Panels

The 41 gene probe panel (ME001B, www.mlpa.com) used in this cohort interrogates 38 unique genes implicated in cancer (24 tumor suppressor genes) for methylation status in two separate reactions (one in the absence of the methyl-sensitive enzyme *Hha*I and one in the presence of the *Hha*I enzyme). There are two probes each for *MLH1*, *RASSF1* and *BRCA2*, and a normal control DNA sample will generate 41 individual peaks in the absence of *Hha*I and 15 individual peaks in the presence of *Hha*I.

### 3.5. Bisulfite Modification and Methylation-Specific Polymerase Chain Reaction (MSP) Assay

**Figure 3.**
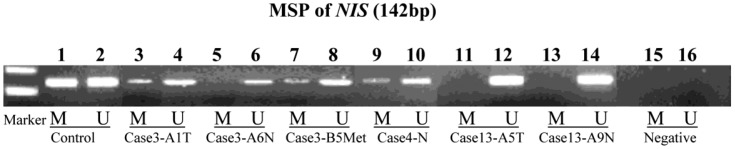
Aberrant methylation of *NIS* detected by methylation-specific PCR (MSP). Lanes 1 and 2: universal methylated and unmethylated controls. Lanes 3-14 span biopsies for Cases 3, 4 and 13. Note presence of methylated product in lanes 3, 7 and 9. Note absence of methylated product in lanes 5, 11 and 13. Lanes 15 and 16: negative control. (A1T – block A1 tumor, A6N – block A6 normal, B5Met – block B5 metastasis, Case4-N – normal thyroid, A5T – block A5 tumor, A9N – block A9 normal).

Genomic DNA (100 ng) from formalin-fixed paraffin embedded normal and cancerous thyroid tissue, control universal methylated DNA (Chamicon International, Inc) and control unmethylated DNA (normal genomic DNA) were modified using the EZ DNA methylation gold kit (Zymo Research, Orange, CA) during which methylated DNA is protected and unmethylated cytosine is converted to uracil [49]. The modified DNA served as a template using primers specific for the methylated or modified unmethylated sequences (Table 1). MSP amplification was performed using 3 μL of bisulfite modified DNA in a final volume of 25 μL using Promega Hot Start Master Mix (Promega Biosystems, Sunnyvale, CA), 0.5 μM primer followed by 95 °C 5 minutes then 40 cycles at 95 °C 40 seconds, 60 °C 40 seconds, 72 °C 1 min [49]. The resultant PCR products were separated on 2% agarose gel stained with ethidium bromide and visualized under UV illumination (Figure 3).

## 4. Conclusions

Our data suggests that aberrant methylation of *CASP8, RASSF1*, and *NIS* maybe an early change in thyroid tumorigenesis regardless of cell type. Epigenetic events of promoter hypermethylation are emerging as promising molecular targets for cancer detection and represent an important tumor-specific marker in tumorigenesis. Further confirmation and validation of these findings in larger cohorts would support these genes as early makers of thyroid tumorigenesis.

## Figures and Tables

**Table 1.** Methylation-Specific PCR (MSP) Primer Sequences for *NIS* gene.

	**Methylation Specific Primers**	**Unmethylation Specific Primers**	**Product Size**
Forward	5′-ATAGGGAGGTCGATACGGATATC	5′-ATAGGGAGGTTGATATGGATATTGA	M-142 bp
Reverse	5′-GAAAAAACAAAACGAAAAAAACG	5′-AAAAAAACAAAACAAAAAAAACAAA	U-142 bp

M = methylated product; U = unmethylated product
